# Could tDCS Be a Potential Performance-Enhancing Tool for Acute Neurocognitive Modulation in eSports? A Perspective Review

**DOI:** 10.3390/ijerph18073678

**Published:** 2021-04-01

**Authors:** Sergio Machado, Bruno Travassos, Diogo S. Teixeira, Filipe Rodrigues, Luis Cid, Diogo Monteiro

**Affiliations:** 1 Laboratory of Physical Activity Neuroscience, Physical Activity Sciences Postgraduate Program, Salgado de Oliveira University, Niterói 24456-570, Brazil; secm80@gmail.com; 2 Department of Sports Science, University of Beira Interior, 6201-001 Covilhã, Portugal; bfrt@ubi.pt; 3 Laboratory of Physical Activity Neuroscience, Neurodiversity Institute, Queimados 26325-020, Brazil; 4 Research Center in Sport, Health and Human Development (CIDESD), 5000-558 Vila Real, Portugal; luiscid@esdrm.ipsantarem.pt; 5 Portugal Football School, Portuguese Football Federation, 1495-433 Cruz Quebrada, Portugal; 6 Faculty of Physical Education and Sport, Lusófona University, 1749-024 Lisbon, Portugal; diogo.sts.teixeira@gmail.com; 7 Research Center in Sport, Physical Education, and Exercise and Health (CIDEFES), (CIDEFES), 1749-024 Lisbon, Portugal; 8 Sport Science School of Rio Maior, ESDRM-IPSantarém, 2040-413 Rio Maio, Portugal; frodrigues@esdrm.ipsantarem.pt; 9 Life Quality Research Center (CIEQV), 2040-413 Rio Maior, Portugal; 10 ESECS, Polytechnic of Leiria, 2411-901 Leiria, Portugal

**Keywords:** dorsolateral prefrontal cortex, eAthletes, eSports, transcranial direct current stimulation

## Abstract

Competitive sports involve physical and cognitive skills. In traditional sports, there is a greater dependence on the development and performance of both motor and cognitive skills, unlike electronic sports (eSports), which depend much more on neurocognitive skills for success. However, little is known about neurocognitive functions and effective strategies designed to develop and optimize neurocognitive performance in eSports athletes. One such strategy is transcranial direct current stimulation (tDCS), characterized as a weak electric current applied on the scalp to induce prolonged changes in cortical excitability. Therefore, our objective is to propose anodal (a)-tDCS as a performance-enhancing tool for neurocognitive functions in eSports. In this manuscript, we discussed the neurocognitive processes that underlie exceptionally skilled performances in eSports and how tDCS could be used for acute modulation of these processes in eSports. Based on the results from tDCS studies in healthy people, professional athletes, and video game players, it seems that tDCS is applied over the left dorsolateral prefrontal cortex (DLPFC) as a potential performance-enhancing tool for neurocognition in eSports.

## 1. Introduction

Electronic sports, better known as eSports, is a worldwide phenomenon, particularly regarding sales and media. The development of professional eSports leagues, the number of spectators following the tournaments, and financial investments increasing exponentially, have solidified eSports in competitive sports culture [1].

Competitive sports involve physical and cognitive skills [2]. However, in more traditional sports, there is a greater dependence on the development and performance of both motor and cognitive skills, unlike eSports athletes (eAthletes), who seem to depend much more on neurocognitive skills for success [3]. 

In line with that, with the growth interest in eSports, players search for ways to improve their performance, through training and using tools that generate a performance advantage [4]. Nowadays, with a growing interest in improving neurocognitive skills, video games and online games seem to be promising in understanding how neurocognitive enhancers can impact competition and performance in eSports [2]. However, despite the competitive nature of eSports, where there are strong mental demands [5], little is known about the neurocognitive functions and effective strategies designed to develop and optimize neurocognitive performance in eSports.

Transcranial direct current stimulation (tDCS) is a well-tolerated, noninvasive brain stimulation technique, characterized by a weak electric current (1–2 mA) applied through electrodes placed on the scalp to induce prolonged changes in cerebral excitability for a long time, even after the end of the stimulation [6]. The anodal current increases the cortical excitability, favoring the depolarization of the neuronal membrane, whereas the cathodal current has an inhibitory effect, causing hyperpolarization of the neuronal membrane [7,8,9]. These effects, depending on the intensity and duration of the electric current imposed through the tDCS, can last for more than an hour [10]. 

Several studies provide good evidence showing that tDCS, mainly anodal tDCS (a-tDCS), directly applied to the left dorsolateral prefrontal cortex (DLPFC), may be a promising performance-enhancing tool for acute modulation of neurocognitive functions in a healthy population [11,12,13,14,15,16,17,18,19,20] and athletes [21,22]. Researchers are starting to examine the effects of tDCS on modulation of neurocognitive processing by eSports athletes (eAthletes) and competitive video game players; however, little is still known about the scientific evidence in this field [4].

Therefore, our aim is to propose a-tDCS as a performance-enhancing tool for neurocognitive functions in eSports, since this modality has been gaining attention [2,3]. We highlighted the relevant role of tDCS in eSports—in likely facilitating neurocognitive skills performance. In addition, we also pointed out safety issues and caveats associated with tDCS use when applied to improve neurocognitive performance for eSports players, as well as ethical, and regulatory aspects.

## 2. eSports-Cognitive Performance Requirements

In recent years, eSports has grown exponentially and has gained great popularity (i.e., socially, and in public media). Playing video games for leisure is not the same as training to compete or to be a professional athlete [4]. eSports is a profession for eAthletes; it even involves training routines (like how athletes in traditional sports participate in training routines), while casual players play for fun [23]. eSports is subdivided into several categories, including online multiplayer, online multiplayer role-playing, real-time strategy, and first-person shooter games. In these types of games, performance is usually carried out as a team, where the player’s avatar plays his role in each virtual environment, to eliminate his competitors, or reach a goal [24,25]. For a player to meet all these specific requirements, the player must mobilize various neurocognitive skills, such as decision-making, anticipation, and attention, among others [26,27].

Even with the growth of eSports, there is still a shortage of research on high-level performance [2]. For example, there are few studies exploring neurocognition in eSports [26,27,28,29], with most papers exploring different aspects of cognition, but in recreational video game players [26,30].

Few studies have investigated the neurocognitive aspects involved in eSports [27,28,29]; thus, the processes underlying performance are still unclear. The characteristics of the environment, in which eSports take place, can offer enhanced ecological validity in research based on traditional sports when exploring specific neurocognitive processes [30,31]. Yet, it is of utmost importance to determine if there is already empirical work testing neurocognitive processes in laboratory settings that could shed light on eSports neurocognitive performance.

Previous research revealed that eSports performance showed high dependency of neurocognitive components [2,32,33]. For example, Bonnar [34] discovered that two of the most important neurocognitive processes involved in eSports are attention and working memory. This is due to the duration of the matches, which can be over 40 min, meaning eAthletes need to mobilize concentration for very long periods to keep the focus on important aspects of the game. For example, the use of selective attention seems to reduce the impact of environmental distractions, such as noise outside the game. Another important factor for eSport performance is executive functioning—that is, cognitive flexibility, problem solving, and decision-making. These functions are fundamental for a better implementation of strategies and tactics to achieve the respective objectives in the game [29].

The study by Li et al. [29] demonstrated that elite League of Legends players were superior to intermediate players in Stroop switching and continuous performance tests, showing better cognitive flexibility and more accurate control of interference in the context of task-switching, in addition to better impulsive control compared to intermediate players. This evidence shows that gaming skills, rather than gaming experience, are more related to neurocognitive functioning. Thus, concerning practical implications, we can propose two possible explanations. First, only players who have better neurocognitive functioning, such as cognitive flexibility, working memory, inhibitory control and decision making, can achieve better rankings or become an elite player. Otherwise, video game training and experience seem not to be the main points; because the best players can simply be the ones with the best neurocognitive functioning. Within this context, Boot et al. [34] argued that playing video games may not improve neurocognitive functioning; and individuals with better neurocognitive functioning may be more likely to play video games because they can perform better than others in the game, reflecting a self-selected process in the video game. The second potential explanation may be related to neurocognitive functions expertise, they can be better trained and enhanced by experience with video game training on players who have a strong motivation to constantly improve their gaming skills and win awards. Mobilization from a variety of neurocognitive resources would be required for an athlete to participate and compete at the highest-level.

## 3. a-tDCS as a Potential Performance-Enhancing Tool for Acute Modulation of Neurocognitive Functions in eSports

Considering the scientific and technological development that have been seen in sports, especially over the past two decades, there is a growing interest by researchers to investigate the potential effects of different resources for improving neurocognitive performance of athletes [2]. Therefore, concerning the effects of tDCS as performance-enhancing tool for acute modulation of neurocognitive functioning in healthy individuals [11,12,13,14,15,16,17,18,19,20], athletes [21,22], and video game players [4], tDCS seems a promising tool for neurocognitive performance in eSports.

tDCS is a non-invasive brain stimulation technique that is considered cheap, safe, painless, and portable. tDCS is composed of a battery-powered stimulator that provides weak electrical currents (0.5–2 mA), using sponges soaked in saline fluid [10]. Generally, brain modulation is dependent on the polarity of the applied current. tDCS allows two types of stimulation: (i) anodal tDCS (a-tDCS), used to stimulate an area of interest, where the anodal electrode is positioned on the target area, while the cathodal electrode acts as the reference electrode to close the electrical circuit, being positioned, in general, over the contralateral supraorbital region or in the deltoid muscle; and (ii) cathodal tDCS (c-tDCS), used to inhibit an area of interest, but with reverse positioning of the electrodes, with the cathodal electrode over the target area and the anodal electrode over the supraorbital region or in the deltoid muscle [10,35,36]. In most studies, the reference electrode was usually placed on the supraorbital region; however, in others, it was positioned over extracephalic regions (e.g., the shoulder). 

**Figure 1:**
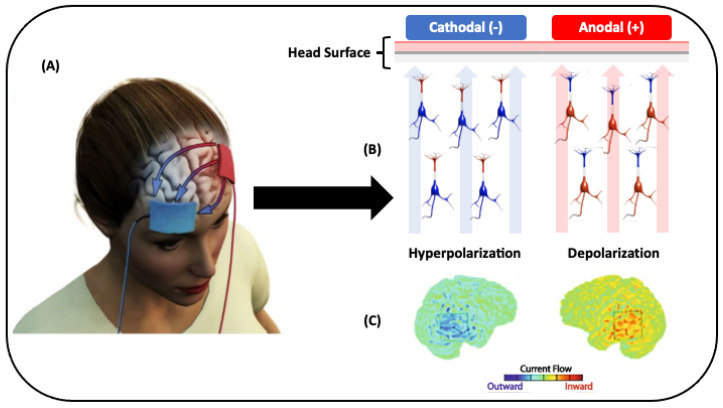
Mechanisms of action induced by transcranial direct current stimulation (tDCS). (**A**) Red electrode (anodal) delivering a weak electrical current towards the blue electrode (cathodal); (**B**) left side (cathodal electrode) shows the hyperpolarization of the neurons, provoking an inhibition in neuronal activity. Right side (anodal electrode) shows the inverse behavior, the depolarization of the neurons, generating an increase in neuronal activity. (**C**) Computational model showing in the left side a brain with reduced activity and, on the right side, a brain with increased activity.

The conditioning effects of tDCS on the rate of neuronal firings have been attributed to changes in the neuronal membrane potential in the stimulated region. a-tDCS is generally known to depolarize neurons, facilitating neuronal firing, whereas c-tDCS generally hyperpolarizes neurons, inhibiting neuronal firing below the stimulation site [10,37] (see Figure 1). Changes caused by tDCS may last beyond the stimulation, if applied for at least three minutes [10], and remain stable for at least one hour if tDCS is applied for a time ≥ of 10 min using current with intensities between 1 and 2 mA [6].

Considering the brain networks involved in neurocognitive processing, one of the most studied areas is the dorsolateral prefrontal cortex (DLPFC), which is responsible for executive control, the ability to orchestrate thoughts and actions in accordance with internal goals [38,39]. Several studies have shown that a-tDCS over the left DLPFC improved acutely the core neurocognitive functions (i.e., working memory, decision making, attention, and multitasking) in healthy people [11,12,13,14,15,16,17,18,19,20], athletes [21,22], and video game players [4]. Therefore, in practical terms, based on the findings from those studies [4,11,12,13,14,15,16,17,18,19,20,21,22], and from safety guidelines [40,41], it seems that a possible strategy of tDCS application in eSports is to use a-tDCS over the left or right DLPFC for 20–30 min, administered at 0.5–2 mA, with the smallest squared electrodes possible (e.g., between 9 and 25 cm^2^), or high-definition tDCS (HD-tDCS), always approximately 20–30 min before training sessions and competitions in order for priming the DLPFC.

The priming effect occurs when an individual is exposure to a certain stimulus (i.e., tDCS) subconsciously, and this stimulus influences the response to a subsequent stimulus (i.e., executive processing) [42]. The idea is to use tDCS as a neuropriming, inducing a temporary state of hyperplasticity in the brain, which would reinforce the ability of the brain to learn, building stronger, and more optimized neural connections for neurocognitive processing [43]. Thus, the hyperplasticity state could allow eAthletes to improve their neurocognitive processing faster.

Following these recommendations, the study of Borduchi et al. [22] was the first until now to apply tDCS as a performance-enhancing tool for neurocognitive processing in professional athletes of judo, swimming, and rhythmic gymnastics. They received a-tDCS administered at 2 mA, with 25 cm^2^ electrodes, for 20 min over left DLPFC, for 10 consecutive weekdays. Athletes improved in alternated, sustained, and divided attention, and in memory performance after receiving a-tDCS compared to sham-tDCS. In another study, Friehs et al. [22] applied a-tDCS and c-tDCS at 0.5 mA, with 9 cm^2^ electrodes, for 19 min over the left DLPFC of basketball players on the head-fake task. When compared to c-tDCS, a-tDCS led to enhanced performance by reducing the interference effect produced by head-fake effect (i.e., interference processing). Moreover, more recently, Friehs et al. [4] delivered a-tDCS at 0.5 mA, with 9 cm^2^ electrodes, for 19 min over the right DLPFC of video game players to investigate effects of a-tDCS on response inhibition in Stop-Signal Reaction Time task, with a significant decrease in SSRT after a-tDCS compared to sham-tDCS. These findings provide preliminary evidence that a-tDCS can be used as an advantageous performance-enhancing tool for acute modulation of neurocognitive functions [4,22], with relevant implications for the real-life application of tDCS as a performance-enhancing tool in professional athletes [21,22] and video game players [4].

Despite the significant effects found on acute modulation of neurocognitive functions in healthy people [11,12,13,14,15,16,17,18,19,20], athletes [21,22] and video game players [4], especially in healthy young adults, tDCS seems a promising performance-enhancing tool for eSports. At this moment, there is no standard protocol or linear dose-response concerning the use of tDCS for modulation of neurocognitive functions. Therefore, the effects depend on many factors that can be optimized. 

Several factors can influence the effects of tDCS, such as whether the stimuli are administered alternately or consecutively [44,45], the use of different tDCS devices, electrode materials, target brain area, [46,47], the distance between the stimulation electrodes (for example, large distances between the electrodes can decrease the magnitude of the effects depending on the assembly used) [48,49], shape (round versus rectangular), and size of the electrodes (25–35 cm^2^), as well as their arrangement in the head [50,51]. In addition, the individual characteristics of the study sample, such as variations in anatomy and physiology can induce very different electrical fields and generate different effects on brain functioning [52,53]. To circumvent these methodological differences, studies have been promoting technological advances that promise, for example, to improve the estimation of electric fields induced by tDCS, to personalize assemblies for brain anatomy individually and to investigate the effects of tDCS on brain physiology. In addition, we believe that researchers should consider the administration of tDCS through a series of smaller electrodes, a technique known as HD-tDCS and, in appropriate circumstances, using assemblies created to use optimization techniques based on standardized brain modeling [54], to create more focal and “personalized” targets of tDCS [47,55,56,57]. The effects of tDCS can also be influenced by the stimulation time—that is, before, during, or after a task [58], whether it is applied in combination with pharmacological manipulations [59], or with a task depending on the type of task used [60], the sensitivity of the measurements before and after the stimulation (especially for healthy people), and the best time for the interval between new stimulation sessions to sustain the results achieved [61].

Other factors that should be highlighted are the basal brain state and its existing connectivity in those who are receiving tDCS. In addition, differences in head size and skull thickness, as well as neuroanatomical differences below the stimulated areas, can affect the distribution of current flow through the cortex [62], raising the question about the need to use neuronavigation. The influence of age has also been reported by studies [63], as well as individual differences, such as basic skills in certain tasks [64], educational background [65], and even personality [66]. Due to all these data regarding the observed relationship between the brain and behavior, new studies could use multimodal neuroimaging techniques to better understand the underlying biochemistry of such interactions. Therefore, we believe that a standardization of these factors, as well as new techniques, will lead to greater and more consistent effects for tDCS intervention.

One of the main sources of this inconsistency is the individual differences between participants, but those differences are rarely examined in the context of combined training/stimulation studies. Studies show that there is a great variability in cortical excitability between individuals, as well as in the response to tDCS, suggesting that stimulation may influence individuals differently, due to age, sex, brain state, hormonal levels, and pre-existing cortical excitability. These findings are extremely important since certain factors can lead to the reversal of polarity-dependent effects [67]. In addition, it is unclear how long the effects of stimulation will finally take, even in successful interventions. Some studies made use of follow-up assessments, but very few have measured performance more than a few months after an intervention.

Although tDCS is not yet approved by the Food and Drug Administration (FDA) for clinical use, and there are no well-established safety guidelines regarding tDCS use [42,43], we believe that tDCS represents a non-significant risk for participants when the recommended procedures are correctly followed [37,68,69]. In line with this, there are risk and safety factors that researchers, professionals, and athletes should be aware of, including the potential long-term adverse effects, effects of prolonged stimulation or repetitive application, and individual response differences (e.g., gender) [70,71]. These questions are important because most players, whether amateurs or professionals, are children and adolescents [72,73], which puts them at greater risk. 

At last, according to the World Anti-Doping Association (WADA), for a substance or method to be considered a “doping”, two of three scientific criteria must be answered positively: first, it has potential beneficial effects on athletic performance; second, it presents potential risks to the health of athletes; and third, it violates the spirit of the sport [74,75,76]. We believe that tDCS use has great potential to improve the acute cognitive performance of eAthletes, based on the results of studies already cited with healthy individuals [11,12,13,14,15,16,17,18,19,20], athletes [21,22], and video game players [4]. We also believe that tDCS represents a non-significant risk for participants when the recommended procedures are correctly followed [68,69]. Thus, tDCS is a potential performance-enhancing tool for neurocognitive performance without posing significant risks to the health of eAthletes. Therefore, determining whether tDCS use is a neuro-doping strategy will ultimately boil down to the challenging ethical question of whether it negatively impacts the spirit of sport and fair competition.

## 4. Conclusions

The use of tDCS as an ergogenic resource has received great attention in recent years (in sports sciences and physical exercise), with results showing more evidence of its performance benefits. These results are important because they serve as an impetus to explore the potential of tDCS as a performance-enhancing tool for neurocognition in eSports. Within this context, based on some studies about the effects of tDCS on modulation of acute neurocognitive processing in healthy people, professional athletes, and eAthletes, we suggest that a-tDCS applied over the DLPFC could be considered a potential performance-enhancing tool for acute neurocognitive modulation for eSports performance.

The advantages of tDCS use have been discussed in the literature, such as being easy-to-use, its low price, no severe adverse effects, and its ethical and legal issues—concerning its transition from academic studies to general-public use—with no prohibition (at the moment) from the World Anti-Doping Association (WADA). The disadvantages are the necessity of daily use by users and its limited lasting effect. Therefore, we highlight the relevance of tDCS for eSports, emphasizing that there are still (several) technical, ethical, and regulatory aspects that must be considered in relation to eSports.
